# The Feasibility and Acceptability of Two Methods of Snack Portion Control in United Kingdom (UK) Preschool Children: Reduction and Replacement

**DOI:** 10.3390/nu10101493

**Published:** 2018-10-12

**Authors:** Sophie Reale, Colette M. Kearney, Marion M. Hetherington, Fiona Croden, Joanne E. Cecil, Sharon A. Carstairs, Barbara J. Rolls, Samantha J. Caton

**Affiliations:** 1 School of Health and Related Research (ScHARR), University of Sheffield, Sheffield S1 4DA, UK; slreale1@sheffield.ac.uk (S.R.); c.kearney@sheffield.ac.uk (C.M.K.); 2 School of Psychology, University of Leeds, Leeds LS2 9JT, UK; m.hetherington@leeds.ac.uk (M.M.H.); f.c.croden@leeds.ac.uk (F.C.); 3 Population and Behavioural Sciences, School of Medicine, University of St Andrews, St Andrews KY16 9TF, UK; jc100@st-andrews.ac.uk (J.E.C.); sc295@st-andrews.ac.uk (S.A.C.); 4 Department of Nutritional Sciences, College of Health and Human Development, The Pennsylvania State University, University Park, PA 16802, USA; bjr4@psu.edu

**Keywords:** portion size, snack reduction, snack replacement, preschool children, feasibility, acceptability

## Abstract

Large portions of high energy dense (HED) snacks are offered to children from a young age and are pervasive in our food environment. This study aimed to explore the feasibility, acceptability, and preliminary efficacy of two strategies of snack portion control: reduction and replacement. Forty-six mother-child dyads aged 22–56 months (36.6 ± 9.5 m, 48% female) completed a three-week intervention. In week 1 (baseline) no changes were made to the child’s diet; week 2 (acclimation) children received a standardised selection of HED snacks, and in week 3 (intervention) participants were randomly assigned to snack replacement (*n* = 24) or snack reduction (*n* = 22). Snack replacement involved swapping HED snacks for fruits and vegetables, whilst snack reduction involved reducing the size of HED snacks by 50%. Food and energy intake were measured using a weighed food diary for four consecutive days. Snack replacement resulted in more positive changes to children’s diets; vegetable intake increased (*p* < 0.01), and total daily energy intake decreased when compared to snack reduction (*p* < 0.05). Mothers expressed a more favourable attitude to snack replacement, although snack reduction was also well received by mothers. Despite increased preliminary efficacy of snack replacement on dietary intake, both strategies were feasible and acceptable. The current pilot study provides the necessary information to inform the design of future interventions.

## 1. Introduction

Despite efforts to address poor dietary intake, children’s diets remain less than nutritionally optimal with many young children consuming diets that contain excessive amounts of energy, salt, sugar, and low intakes of fruit and vegetables [1]. It is known that poor diet quality and excess energy intake relative to expenditure contribute to the development of chronic diseases in adulthood [2]. Dietary habits established early in life track into later life [3], highlighting the importance of establishing healthy eating in the early years. During early childhood, the family environment is one of the main influencing factors on diet quality [4]. There is a positive relationship between maternal and child intake for core and non-core/snack food items [5] and a similar relationship for portion sizes served [6]. Eating between-meal snacks in young children may be necessary to support growth and development [7]. However, data from the United States (US), Canada, and Europe suggest that snack foods contribute a significant amount of energy, salt, and sugar to the habitual diet [8,9,10]. In the present study, we tested two strategies to modify snacking behaviour that have the potential to improve children’s diets.

High energy dense (HED; >2.5 kcal/g) foods [11] including many snacks are thought to contribute to excess energy intake and increase the risk of overweight/obesity in paediatric populations [12,13,14]. Snacking has also been related to poor diet quality [7,15]. In the United Kingdom (UK) half of the sugar children consume is derived from HED snacks, such as confectionary (sweets and chocolate), cakes, buns, biscuits and sugary drinks [1]. A recent survey carried out by the Infant and Toddler Forum [16] reported that children as young as age two are being offered large, adult-sized portions of HED snacks. Similarly, in the US, 57% of preschool children are consuming cookies and candy daily [17]. A study examining US preschoolers aged 2–5 years demonstrated that the frequency of snacking and body weight are positively related [18].

In 2018, Public Health England (PHE) launched the campaign “*Look for 100 calorie snacks, two a day max*” advising caregivers to limit the frequency and energy content of children’s snacks to twice a day, with a maximum of 100 kcal per snack [19]. This campaign was launched as a bid to reduce sugar intake at the population level. This advice contributes towards efforts to reduce total daily energy intake (TDEI) from free sugars by 50%, as recommended by the World Health Organisation (WHO) [20] and dietary guidelines in the US [21]. However, given that most adults and children are exceeding 11% of their TDEI from sugar, reducing this to 5%, as recommended by the UK Scientific Advisory Committee on Nutrition [22], constitutes a significant and challenging shift in dietary behaviours. Smaller portion sizes of HED snack foods might facilitate the consumer’s ability to achieve this target.

Parents are known to make a judgement regarding portion size based on package labelling [23] or how much they serve themselves [6]. Thus, one possible approach to portion size reduction could be the provision of smaller snacks. Since many snacks are offered according to package size, a simple message to caregivers might be to split the “usual” snack in half [23]. Alternatively, caregivers could be instructed to replace HED snacks with liked and familiar fresh fruit and vegetables since children may accept these foods as alternatives [24,25]. Repeated exposure increases food acceptance and preference [24], and therefore replacing HED with low energy dense (LED; <1.51 kcal/g) snacks [11] may be a potential strategy to encourage sustained improvements in children’s diets. Little is known about the effects on children’s habitual diets of reducing or replacing HED snacks with those lower in energy density. A US-based study [26] that was carried out in a child care setting examined the effects of offering preschool children fruit and vegetables as snacks. Whilst the children demonstrated a preference for fruit overall, offering vegetables as snacks increased intake of vegetables as well as fruit [25]. Whilst offering vegetables as a snack seems to increase vegetable intake, we do not know whether children offered vegetables as snacks compensate by reducing their vegetable intake elsewhere in the diet, for example, at meals.

Before conducting a randomized clinical trial to evaluate strategies to manage children’s portions of HED snacks, it is advisable to test feasibility and acceptability. The National Institute for Health Research (NIHR) Evaluation, Trials, and Studies Coordinating Centre defines feasibility as an important parameter that is used to design a full-scale study e.g., recruitment, retention, participant eligibility, and compliance [27], whereas acceptability refers to “*judgements by lay persons, clients and others of whether treatment procedures are appropriate, fair, and reasonable for the problem or client*” [28]. Therefore, the aim of the current pilot study was to explore the feasibility and acceptability of two strategies of snack portion control: snack reduction (reducing snack intake by 50%) and snack replacement (replacing all HED snacks with LED fresh fruits and vegetables). The secondary aim was to examine the efficacy of the two methods of portion size reduction to improve the diets of preschool children. For this research, snacking was defined as any food consumed not part of a main meal (breakfast, lunch, or dinner).

## 2. Materials and Methods

### 2.1. Participants

Participants were mothers of children aged 22–56 months who reported primary responsibility for feeding their child. Mothers were recruited from 38 toddler groups across Sheffield once contact and rapport had been made with toddler group leaders. Furthermore, advertisements were posted online (e.g., toddler group websites or Facebook pages) between April and July 2016. Taking into account both the practicality of recruitment and the potential drop-out rates, this pilot study aimed to recruit at least 9% (*n* = 46) of the sample size required to carry out a full trial [29], with a minimum of 12 per group [30]. Inclusion criteria included; parental age of at least 18 years, a commitment to study involvement for three consecutive weeks and consumption of at least one HED commercially available snack per day, as part of the child’s habitual diet. Furthermore, the child had to moderately like and be familiar with the snack items provided. Mothers were excluded if their child had a food allergy or were taking medication known to impact appetite. Due to the requirement of a four-day consecutive food diary (including at least one weekend day), mothers were also excluded if their child attended nursery for more than three full consecutive days. The study was reviewed and approved by the School of Health and Related Research Ethics committee at the University of Sheffield (#007850) and was registered as a clinical trial (#NCT03339986, https://clinicaltrials.gov/ct2/show/NCT03339986). Mothers gave written, informed consent and they were provided with £25 for their time. All foods for the study were provided free to mothers.

### 2.2. Design

A mixed methods approach was taken to provide flexibility and integrity to address the range of research questions [31]. A between subjects three-week intervention was employed with participants acting as their own controls during baseline (week 1) and acclimation (week 2) periods before being randomised to either snack reduction or snack replacement (week 3). Participants were randomised in blocks of ten, to ensure a balanced sample size across treatment groups [32]. The study took place within the home to enhance ecological validity [33]. Each week mothers were asked to keep a four-day weighed food and beverage diary and were provided with snacking schedules to follow in week 2 and 3. Feasibility and acceptability were measured to estimate parameters needed for a full trial, such as participant eligibility, participation rates, compliance, and willingness to continue [34]. Feasibility was measured by exploring retention rates, participation, and compliance. Acceptability was explored via a post intervention questionnaire and semi-structured interviews.

### 2.3. Procedure

Eligible participants, identified from the screening questionnaire, were instructed to keep a weighed food and drink diary, for their child, for four consecutive days. Weighing scales (Salter Essentials Bowl Scale), detailed instructions, photographic examples and a demonstration on how to use the scales accurately was provided. Where possible, mothers were asked to partake in the study on weeks where children were not attending parties. All foods and beverages consumed by their child inside and outside of the home were included, without making any changes to their habitual diet. The researchers (Sophie Reale, Colette Kearney) visited participants prior to weeks 2 and 3 to deliver all food items that were required for study participation.

In week 2, mothers were instructed to replace HED snacks with snacks in the snacking schedule, at their child’s usual snack time. Mothers were instructed to provide the usual amount (1, 2, or 3 snacks) and to continue providing fresh fruit and vegetables as part of the habitual diet. However, if they normally provided dried fruit they were advised to replace this with a snack from the schedule given the energy density of dried fruit (e.g., raisins).

In week 3, mothers were randomly allocated to the snack reduction or snack replacement condition via a simple randomisation procedure (block randomisation). In both conditions, mothers were provided with a range of snacks that were intended to replace all HED snacks usually consumed (Table 1). Snack types, amounts, and quantity offered per day were chosen based on data collected from the cohort prior to the beginning of the experiment, when caregivers expressed an interest in participating (data not shown). To ensure that all children received the same selection and quantity of snacks, snack schedules were devised (week 2 and 3), providing up to three snacks a day for 7 days. There were no snack repetitions in a day, so each child regardless of whether they had 1, 2, or 3 snacks per day could be offered each snack item at least once per week. Those in the reduction condition received the same HED snacks as week 2, but were instructed to provide a 50% portion at each snack occasion. Full portions were provided to allow mothers to decide how to serve the half portion (e.g., in the original packaging or on a plate/bowl). In the replacement condition, mothers were instructed to remove all HED snacks and sugar sweetened beverages from their child’s diet and offer 40 g of fresh fruits and 40 g vegetables, a starch-based food (bread stick, rice cake, or crackerbread) served together, and no-sugar alternative drinks (see Table 1). In both conditions, zip lock bags and food clips were provided to store left over food items and enable snacks to be consumed outside of the home or saved for a later snacking occasion. If the child was still hungry after the snack offering, caregivers were advised to provide more of the fresh fruit or vegetable components.

At the end of the intervention, mothers were invited to complete the acceptability questionnaire and the follow-up questionnaire, 4–6 weeks post intervention. A random sample of mothers *n* = 26 (*n* = 13 reduction/13 replacement) were also invited to participate in a short semi-structured interview to explore, in more detail, the feasibility and acceptability of the intervention. Questions were related to the ease of completing the food diaries and how the child responded to the intervention. The number of interviews conducted was determined by the point at which theoretical saturation was achieved [35]. All of the interviews took place within the family home between December 2016 and May 2017, and were audio recorded. Each interview lasted around 30 min and took place immediately post intervention.

### 2.4. Materials and Measures

#### 2.4.1. Anthropometrics

All children’s heights (m) and weights (kg) were measured by the researcher. Weights were measured using digital scales (Seca, Hamburg, Germany) and height measured using a portable stadiometer (Seca SMSSE-0260, Leicester, UK). Weight-for-height z-scores were calculated using the WHO anthropometric calculator (http://www.who.int/childgrowth/software/en/).

#### 2.4.2. Screening Questionnaire

The screening questionnaire collected demographic data on child age, gender, parental age, body mass index (BMI) (self-report height and weight), income, education, employment, ethnicity, accommodation status, and marital status. Information regarding child care (day per week), food allergies, and whether the child liked and regularly consumed HED snacks, fruits, and vegetables was also collected to establish typical patterns of food intake. This information was collated by the primary researcher to ensure that participants met inclusion criteria.

#### 2.4.3. Food Frequency Questionnaire (FFQ)

A shortened version of the FFQ [36] containing snack items (sweet biscuits, cakes/scones, sweet pastries, sweets/chocolate bars, crisps, green cooked vegetables, other cooked vegetables, salad, fresh fruit) was administered to mothers during recruitment to determine eligibility to take part. The same shortened FFQ was administered to mothers 4–6 weeks post intervention to identify any longer-term changes to child intake of fruit, vegetables, and HED confectionary/snacks.

#### 2.4.4. Parent and Child Characteristics

Information regarding child individual characteristics was collected to examine the potential differences between groups. Several validated questionnaires were administered to mothers to provide an overview of child eating traits and parental feeding practices. These included; the Children’s Feeding Practice Questionnaire (CFPQ) [37], the Child Eating Behaviour Questionnaire (CEBQ) [38], and the child food neophobia scale [39] as child neophobia has been linked to lower intakes of fruits and vegetables [40]. Furthermore, impulsivity and inhibitory control have been associated with overweight and obesity [41,42], and so the relevant items from the Early Childhood Behaviour Questionnaire (ECBQ) [43] were included.

#### 2.4.5. Acceptability Questionnaire

An 18-item questionnaire was developed based on previous work [44,45] to explore the acceptability of the study procedures, the types and amounts of snacks provided, and the longer-term engagement with the intervention. Each question was scored on a five-point Likert scale ranging from “strongly disagree–strongly agree” “very unlikely/unwilling–very likely/willing” (see Table A1).

#### 2.4.6. Follow-Up Questionnaire

The follow-up questionnaire was administered 4–6 weeks post intervention. It comprised of the FFQ [36] and three open-ended questions regarding the child’s current snack intake and familial eating habits. For example, “*Has taking part in the study had any impact on your child’s snack intake/overall diet? If yes, how? If no, why not?*”, “*Has taking part in the study had any impact on other members of the family? If yes, how? If no, why not?*”.

#### 2.4.7. Food Diary

Mothers completed weighed food diaries to assess their child’s food (meals and snacks) and beverage consumption and to provide information regarding portions eaten. In line with previous interventions, and to reduce participant fatigue, four consecutive days were recorded, including at least one weekend day [1].

### 2.5. Data Analysis

#### 2.5.1. Qualitative

Qualitative data (semi-structured interviews and responses to open ended questions from the follow-up questionnaire) were transcribed verbatim and they were collated into NVivo for thematic analysis (by SR). As part of Braun and Clarke’s [46] six phase process of thematic analysis, codes were initially generated by reading each transcript line by line. Data was coded inclusively (text before and after the section of interest was coded) to maintain context throughout the analysis, and in some instances, segments of data were coded multiple times due to their relevance to multiple codes. The generated codes were organised into broader themes by collating related codes. An inductive approach was taken; ensuring themes were strongly related to the data itself [47] rather than the researcher’s theoretical or analytic interests [48]. Each phase of Braun and Clarke’s [46] guidelines was applied as part of a recursive process, as the analysis developed over time [49]. Ten percent of manuscripts were crosschecked by a second reviewer (CK). Discrepancies were discussed until consensus was achieved.

#### 2.5.2. Quantitative

All quantitative analyses were carried out using SPSS (IBM SPSS Statistics v22, Armonk, NY, USA). Data are presented as mean (±SD) and percentages. Inferential statistics were used to examine feasibility (participation and compliance), acceptability, retention, preliminary efficacy of each intervention on dietary intake, and predictors of vegetable intake. Participation was recorded as the number of days’ mothers completed the food diary in weeks 1, 2, and 3. Compliance was defined as the percentage of food diary days where mothers followed the snacking schedules in week 2 and 3. Each day was examined individually and recorded as a compliant or non-compliant day. Days were coded as compliant when the mother had provided at least one scheduled snack, and no additional snacks, other than fresh fruit and vegetables. Days were coded as non-compliant if the child had not been offered a snack from the schedule, was provided one or more additional snacks not on the schedule, or, when in the reduction condition, a full portion was provided instead of a 50% portion. Individuals were placed in low, medium, and high compliance categories, depending upon whether they complied <50%, 50–74%, or ≥75% of the time. Pearson’s chi square tests were used to identify if there were differences in compliance and acceptability between intervention groups.

Repeated measures ANOVA were used to examine the effect of intervention on dietary intake. Study week was the within subject’s variable and intervention group the between groups variable. Outcome measures were mean consumption per day of vegetables (g), fresh fruit (g), total daily energy intake (kcal), total sugar (g), free sugars (g), total fat (g), and mean number of snacks. For fresh fruit and vegetables, the average intakes were calculated from snacks, meals and total (snacks and meals combined). Where Mauchly’s Test of Sphericity was violated, Greenhouse-Geisser was reported [50]. Where significant interactions were found, graphical representation was used to identify suitable follow up tests. This included using one-way repeated measure ANOVA to identify within subject differences and independent t-test to examine between subject differences. Alpha was set at *p* < 0.05.

Paired sample t-tests were used to examine differences in mean frequency of consumption pre and post intervention (cookies, cakes, pastries, sweets, crisps, green cooked vegetables, other vegetables, salad, and fresh fruit).

Linear regression analyses were performed to identify factors that predicted total vegetable intake, total fruit intake, total energy intake, total fat intake, and total sugar intake in week 3. Twelve variables were included in the initial models (intervention group, baseline intake, child age, child BMI, food fussiness, pressure to eat, food responsiveness, satiety responsiveness, child food neophobia, monitoring, modelling, and deprivation score) as their influential effects on nutritional intake have been discussed in the literature [40,51,52,53]. As part of an automatic procedure, the weakest correlated variable was removed, and a new model created [50]. This process continued until the final models contained only the variables that best explained the distribution in vegetable, fruit, energy, fat, or sugar intake.

## 3. Results

### 3.1. Demographics

A total of 46 mother-child dyads from Sheffield (South Yorkshire, UK) completed the study between December 2016 and July 2017, thus achieving the target sample size. The mean age of the children was 36.6 ± 9.5 months (52% male). They were from mixed socioeconomic backgrounds (46.6% residing in the 50% most deprived areas of the city) with over a quarter of families earning below the average household income for 2017 [54]. Most of the sample were white British, mixed or other (93.5%) and had normal weight status (self-reported). There were no significant differences in children’s eating behaviours or parental feeding practices between conditions (*p* > 0.05). Participant demographics are presented in Table 2.

### 3.2. Participant Recruitment and Retention

**Figure 1:**
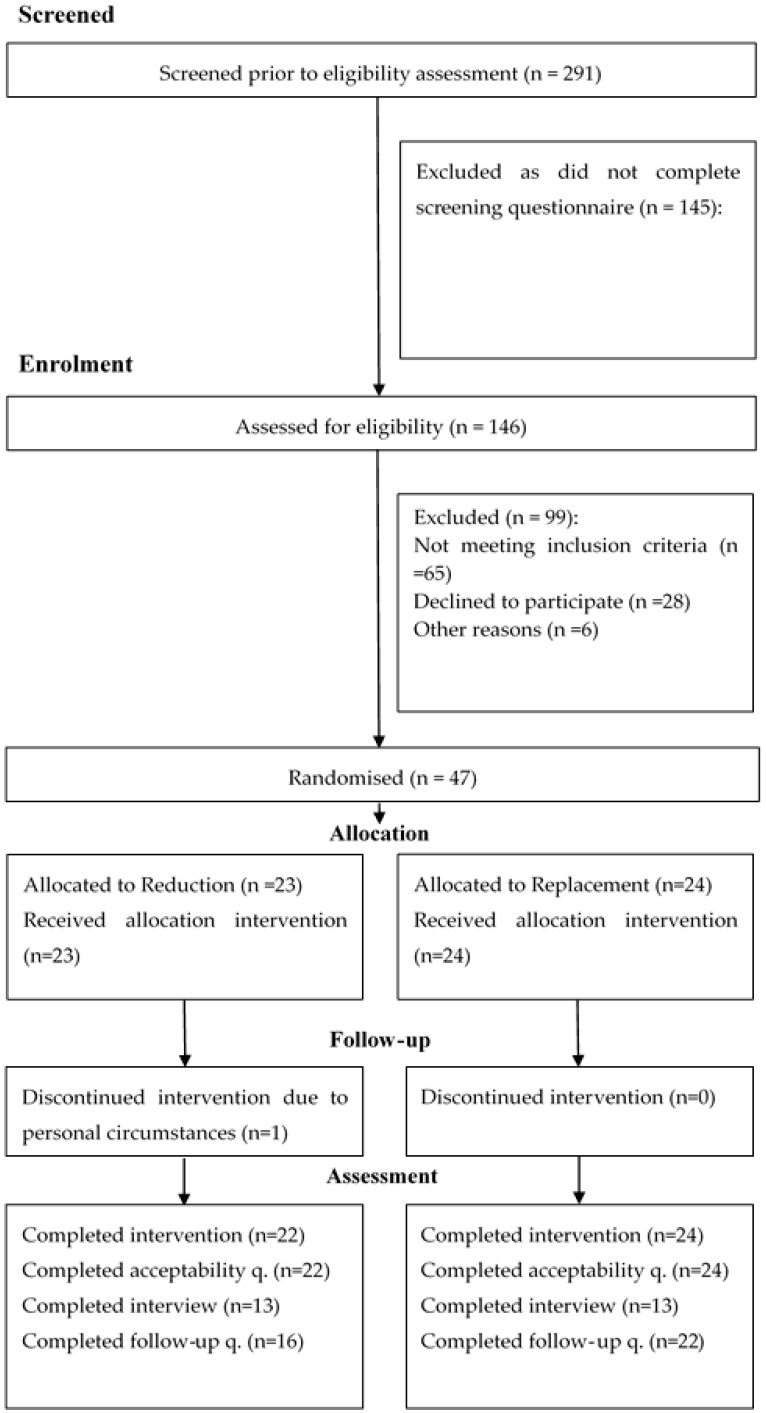
Recruitment and retention rates in accordance to CONSORT guidelines [55].

In total, 291 caregivers expressed an interest in participating in the study and they were sent the screening questionnaire (Figure 1). One hundred and forty-six potential caregivers completed and returned the screening questionnaire and their responses were screened against the inclusion and exclusion criteria. Ninety-nine caregivers (68%) were excluded due to not meeting inclusion criteria (for example, no HED snacks were reported to be habitually offered to children), declining participation, or personal circumstances. The remaining 47 mothers (32%) were eligible and were thus contacted to arrange a date to begin the study. In all, 98% of the 47 mothers (*n* = 46) completed the full three-week intervention, demonstrating excellent retention. Over half of the sample completed a semi-structured interview (*n* = 26) and/or the follow-up questionnaire (*n* = 38) 4–6 weeks post intervention.

### 3.3. Feasibility

Participation remained high across weeks 1 (100%), 2 (100%), and 3 (98%, one mother failed to return the final food diary and was therefore excluded from all diary analyses).

Across the entire study, 22 mothers complied with the snacking schedule on ≥75% of food diary days. Eleven complied on 50–74% of food diary days, whereas 13 complied <50% of the time. Total compliance was associated with study week (x^2^(4) = 22.89, *p* < 0.001) but not condition, (x^2^(2) = 1.70, *p* > 0.05). Compliance to the snacking schedules was higher in week 3 when compared to week 2.

Mothers spoke openly in interviews about why they did not comply with the schedule. Their reasons were categorised into three subthemes: child in the care of others, child health and behaviour, and maternal organisation (Table A2).

#### 3.3.1. Theme 1: In the Care of Others

When children were in the care of others, some mothers were unable to comply with the schedule due to nursery rules regarding the type of snacks that were permitted. Some mothers withheld the snacks to prevent their child feeling isolated or to ensure that other children did not see, and therefore want the snacks their child was consuming, as it was not possible to provide all of their nursery peers with an identical snack option.

When children were in the care of their father or grandparents, occasionally the snacking schedule was not followed. At times caregivers did not follow instructions; at other times mothers expressed a fear of placing pressure on others such as their relatives so they did not ask them to follow the schedule. “*I felt like sometimes I didn’t want to put too much imposition on them, I felt sorry for my mother-in-law having to deal with him screaming*” (P214, Replacement, male, 30 months).

#### 3.3.2. Theme 2: Children’s Health and Behaviour

When children were unwell, mothers appeared more concerned about whether their child was eating as opposed to what they were eating; therefore, the schedule was not followed during times of illness. During illness children were given autonomy in deciding what they wanted to eat. Children were also allowed to choose what they wanted to eat when they were upset, disliked a snack, or simply requested a different one. However, in most cases, it was clear that when some children requested different snacks or refused to eat what was provided, mothers did not accept their child’s requests, as they were determined to comply with the snacking schedules. “*I just stuck to my guns and said no you’re not having it. I mean it’s hard at the time but I stuck to my guns*” (P34, Replacement, female, 48 months).

#### 3.3.3. Theme 3: Maternal Organisation

Mothers who described themselves as organised had no problems following the schedules as they prepared the snacks in advance of their offering. However, in the reduction condition some mothers were less organised and forgot to reduce the snack portion size they offered to their child by 50%. Instead, they tried to remove half once it had been served or allowed their child to consume the full portion. “*I was quite often forgetting to give half. With Pom-Bears (chips) I gave her the pack forgetting that it should be half.*” (P84, Reduction, female, 37 months).

### 3.4. Acceptability

#### 3.4.1. Recording in the Food Diary

Most mothers (76%) reported that recording in the food diary was not a difficult or burdensome task but instead found the food diary a helpful tool. In some, but very few, cases (11%) mothers served their children food items that made record keeping easier. For example, providing a ready meal with predefined weights for each ingredient included.

#### 3.4.2. Week 2 Snacks

Most of the participants agreed/strongly agreed that the snacks provided in week 2 were appropriate for their child (85%), similar to their habitual intake (67%) and liked by the child (87%).

#### 3.4.3. Week 3 Snacks

Most parents (*n* = 31) reported that their child’s hunger was satisfied by the snacks that were provided in week 3, and that the children (*n* = 37) were overall happy with the snacks that they received. Chi square tests revealed no differences between condition and hunger satisfaction, (x^2^(4) = 3.36, *p* > 0.05), however there was a significant difference between the intervention group and children’s perceived happiness with the snacks that they received (x^2^(4) = 13.73, *p* < 0.05). More children in the reduction condition were reported to be happy with the snacks that they received compared to children in the replacement condition (95% vs. 67%, respectively).

#### 3.4.4. Sustainability of the Intervention

Most participants (74%) expressed an interest in continuing with the intervention in the long term. There were mixed views on the likelihood of the intervention making permanent changes to their child’s diets. In the replacement condition, 21% of mothers reported that the intervention was very likely to result in permanent changes to the child’s diet. Similar responses were recorded in the reduction condition (18%). Chi square revealed no difference by condition for the reported likelihood of the intervention making permanent changes to the child’s diet (x^2^(3) = 6.43, *p* > 0.05). However, a significant difference by condition for willingness to continue with the intervention (x^2^(3) = 9.46, *p* < 0.05) was identified. More mothers (92%) were willing to continue replacing their child’s HED snacks with fresh fruit and vegetables than mothers (50%) willing to continue providing smaller portion sizes of HED snacks.

Qualitative responses regarding the acceptability of the intervention were categorised into four subthemes: recording in the food diary, snack type, snack preparation and serving method, and willingness to continue (Table A2).

#### 3.4.5. Theme 1: Recording in the Food Diary

Mothers reported that they felt well equipped to record in the food diary as they had been provided with clear instructions, examples, and weighing scales. They described the food diary as a task that got easier over time and became part of their routine. Mothers found it easy when they were at home with their child, had the scales at hand and recorded in the diary after each eating/drinking occasion, as requested. However, they reported that it was more difficult when they were out of the home. Overall, mothers reported the food diary as a useful tool to see how much their children had consumed over each day and each week. “*Easy, it was easy peasy. I just got it into my routine. I just wrote it every time, every meal, I wrote everything straight away, I weighed it, wrote it down, served it and then weighed what was left.*” (P33, Reduction, female, 39 months).

#### 3.4.6. Theme 2: Snack Type

Mothers discussed the similarities and differences of the snacks provided in the snacking schedules. Most, felt that the week 2 snacks were similar to their usual snack offerings, well liked and suitable for their children. When children liked and were familiar with the snacks, they accepted the changes made. However, when children reported that they disliked the snacks on offer, they were less accepting and sometimes refused to eat. “*I don’t think she cared really actually as long as she likes it she’ll eat it. She wasn’t asking for anything any different.*” (P160, Replacement, female, 28 months).

#### 3.4.7. Theme 3: Snack Preparation and Serving Method

Mothers discussed the impact of preparing and providing the scheduled snacks on their daily routine. The packaged snacks in week 2 were described as convenient and non-disruptive. Mothers reported few problems providing a 50% portion in the reduction condition and most parents were happy to prepare fresh fruits and vegetables for their children. However, they felt that more weighing and preparation was required in the replacement condition when compared to week 2, though this was not perceived as a real burden. “*It was obviously a little bit more faffy than the other one because you have to weigh it, erm, washing it and prepping it before you go out and stuff like that*” (P132, Replacement, male, 45 months).

In the reduction condition, mothers felt that the snack serving method influenced their child’s awareness of the snack reduction and therefore their acceptability of a smaller snack. Mothers who provided snacks on a plate/bowl found that their child did not notice the reduced portion and accepted the snack change. However, children who received the snack in its original packaging often noticed the reduced portion size and requested the rest of the pack. “*Like the crisps maybe I put them in a bowl or something like that so maybe that’s why she didn’t notice as much*” (P20, Reduction, female, 52 months).

#### 3.4.8. Theme 4: Willingness to Continue with the Intervention

Most parents expressed an interest in continuing to use the methods of replacement or reduction when serving their children habitually consumed snacks, as they thought it was an acceptable method of snack portion control in the home environment. “*I will be carrying on and giving her, I’ll mix it all up and make sure I am offering more fruit and veg snacks definitely*” (P77, Replacement, female, 49 months).

### 3.5. Preliminary Effects of the Intervention

#### 3.5.1. Vegetable Intake

1. Vegetables as snacks

Repeated measures ANOVA revealed a main effect of week (F(1.08, 47.40) = 16.37, *p* = 0.00, *η_p_*^2^ = 0.27), and intervention group (F(1, 44) = 14.74, *p* < 0.001, *η_p_*^2^ = 0.25) on vegetable snack intake. Overall in week 3, children consumed 9.8 ± 2.3 (*p* < 0.001) and 9.7 ± 2.4 g (*p* < 0.001) more vegetable snacks when compared to week 1 and 2, respectively. Overall, children in the replacement group consumed 6.1 ± 1.6 g (*p* < 0.001) more vegetable snacks than children in the reduction group. A significant interaction between study week and intervention group was also found for vegetable snack intake (F(1.08, 47.40) = 20.03, *p* < 0.001, *η_p_*^2^ = 0.31). One-way ANOVA identified a significant effect of study week in the replacement group (F(1.02, 23.39) = 20.70, *p* < 0.001, *η_p_*^2^ = 0.47), children in the replacement condition consumed 20.8 ± 4.5 g (*p* < 0.001) and 20.4 ± 4.4 g (*p* < 0.001) more vegetable snacks in week 3 when compared to week 1 and 2, respectively. In week 3, there was also a significant difference in vegetable snack intake between intervention groups (t(23.35) = 4.59, *p* < 0.001, *r* = 0.69). Children in the replacement group consumed 20.5 ± 4.5 g more vegetable snacks per day than the reduction group (Table 3).

2. Vegetables consumed as part of meals only

Vegetable intake at meal times did not vary across weeks or between groups (Table 3). Repeated measure ANOVA revealed no main effect of week (F(2, 88) = 1.91, *p* = 0.15, *η_p_*^2^ = 0.04), intervention group (F(1, 44) = 0.59, *p* = 0.45, *η_p_*^2^ = 0.01), or interaction effect (F(2, 88) = 0.02, *p* = 0.98, *η_p_*^2^ = 0.001) (Table 3).

#### 3.5.2. Fruit Intake

1. Fruit snacks

Repeated measures ANOVA revealed a main effect of week (F(2, 88) = 8.66, *p* < 0.001, *η_p_*^2^ = 0.16) on fruit snack intake. Intake of fruit snacks declined in week 2 as compared to weeks 1 and 3. Pairwise comparisons revealed that in week 2 children consumed 17.4 ± 38.0 g (*p* = 0.035) and 29.3 ± 6.4 g (*p* < 0.001) less fruit snacks compared to week 1 and 3, respectively. There was no main effect of the intervention group (F(1, 44) = 1.63, *p* = 0.21, *η_p_*^2^ = 0.04) and no interaction (F(2, 88) = 1.45, *p* = 0.24, *η_p_*^2^ = 0.03) (Table 3).

2. Fruit consumed as part of a meal

Similar to the results for vegetables, fruit intake as part of a meal did not vary across weeks or between intervention groups (Table 3). No main effect of week (F(2, 88) = 0.10, *p* = 0.91, *η_p_*^2^ = 0.002), intervention group (F(1, 44) = 0.03, *p* = 0.85, *η_p_*^2^ = 0.001), or interaction (F(2, 88) = 0.12, *p* = 0.89, *η_p_*^2^ = 0.003) (Table 3) was found.

#### 3.5.3. Energy (Mean Intake kcal/day)

Repeated measures ANOVA revealed no main effect of week (F(2, 88) = 2.51, *p* = 0.09, *η_p_*^2^ = 0.05) or intervention group (F(1, 44) = 0.07, *p* = 0.79, *η_p_*^2^ = 0.002) on total energy intake per day. However, there was a significant interaction between study week and intervention group (F(2, 88) = 3.18, *p* = 0.047, *η_p_*^2^ = 0.07). A one way repeated measure ANOVA demonstrated a significant effect of study week in the replacement group (F(2, 46) = 5.40, *p* = 0.008, *η_p_*^2^ = 0.19). In week 3, children in the replacement group consumed 145 ± 43 kcal/day (*p* = 0.003) and 87 ± 40 kcal/day (*p* = 0.04) less total energy intake per day than in week 1 and 2, respectively (Table 3).

#### 3.5.4. Sugar Intake

1. Total sugar

A main effect of week (F(2, 88) = 5.12, *p* = 0.008, *η_p_*^2^ = 0.10) was found for total sugar intake. Pairwise comparisons revealed that in week 3 children consumed 10.32 ± 3.17 g less sugar per day than in week 1 (*p* = 0.002). There was no main effect of intervention group (F(1, 44) = 0.04, *p* = 0.84, *η_p_*^2^ = 0.001) or interaction between group and week (F(2, 88) = 2.28, *p* = 0.11, *η_p_*^2^ = 0.05) (Table 3).

2. Free Sugar

A main effect of week (F(2, 88) = 9.06, *p* = 0.00, *η_p_*^2^ = 0.17) on free sugar intake was found. Overall free sugar consumption was the lowest in week 3. Pairwise comparisons revealed that in week 1 children consumed 9.7 ± 3.2 g (*p* = 0.007) and 11.8 ± 2.7 g (*p* < 0.001) more free sugar as compared to week 2 and 3, respectively. No main effect of intervention group (F(1, 44) = 1.75, *p* = 0.19, *η_p_*^2^ = 0.04) or interaction (F(2, 88) = 1.18, *p* = 0.31, *η_p_*^2^ = 0.03) was observed (Table 3).

#### 3.5.5. Fat Intake

A main effect of week (F(2, 88) = 3.30, *p* = 0.04, *η_p_*^2^ = 0.07) on total fat intake was found. Pairwise comparisons revealed that in week 3 children consumed 4.3 ± 1.7 g less total fat per day than in week 2 (*p* = 0.01). There was no main effect of intervention group (F(1, 44) = 0.21, *p* = 0.65, *η_p_*^2^ = 0.005), however a significant interaction between group and week was observed (F(2, 88) = 5.50, *p* = 0.006, *η_p_*^2^ = 0.11). A one way repeated measures ANOVA identified a significant effect of study week in the replacement group (F(2, 46) = 7.42, *p* = 0.002, *η_p_*^2^ = 0.24). In the replacement condition, children consumed 7.5 ± 2.0 g (*p* = 0.001) and 7.8 ± 2.2 g (*p* = 0.002) less fat in week 3, as compared to week 1 and 2, respectively (Table 3).

#### 3.5.6. Mean Number of Snacks (LED and HED) Consumed per Day

A main effect of study week (F(2, 88) = 9.41, *p* < 0.001, *η_p_*^2^ = 0.18) was found for the number of snacks consumed. In week 1, children consumed almost half a snack less than in week 2 (mean difference = 0.4 ± 0.1 g, *p* = 0.002) and 3 (mean difference = 0.3 ± 0.1 g, *p* = 0.009). No main effect of intervention group (F(1, 44) = 1.09, *p* = 0.30, *η_p_*^2^ = 0.02) or interaction between group and week (F(2, 88) = 2.61, *p* = 0.08, *η_p_*^2^ = 0.06) was observed (Table 3)

#### 3.5.7. Predictors of Nutritional Intake

Linear regression models were calculated to investigate the predictors of total vegetable, total fruit, total energy, total fat, and total sugar intake in week 3 (Table 4). For vegetable intake, the final model was strong, accounting for 65% of variance in vegetable intake (R^2^ = 0.65, F = 17.88, *p* < 0.001). Significant predictors included intervention group (reduction or replacement), baseline vegetable intake, child food neophobia, and deprivation score. Higher intake of vegetables in week 3 were associated with being in the replacement group (*b* = 23.91, *se* = 6.34, *β* = 0.39, *p* = 0.001), higher baseline vegetable intake (*b* = 0.72, *se* = 0.13, *β* = 0.58, *p* < 0.001), higher deprivation score (higher scores indicate lower levels of deprivation) (*b* = 2.06, *se* = 0.98, *β* = 0.21, *p* = 0.04), and lower food neophobia scores (*b* = −1.59, *se* = 0.62, *β* = −0.27, *p* = 0.01).

For fruit intake the final model accounted for 63% of variance (R^2^ = 0.63, F = 7.07, *p* < 0.001). Significant predictors included baseline fruit intake, food fussiness, child food neophobia, and modelling. Non-significant predictors included intervention group (reduction or replacement), food responsiveness, satiety responsiveness, and child BMI centile. Higher intake of fruit in week 3 was associated with a higher baseline fruit intake (*b* = 0.72, *se* = 0.13, *β* = 0.63, *p* < 0.001), higher food fussiness (*b* = 5.84, *se* = 2.56, *β* = 0.53, *p* = 0.03), lower child food neophobia (*b* = −7.99, *se* = 2.21, *β* = −0.77, *p* = 0.01), lower modelling scores (*b* = −10.25, *se* = 2.38, *β* = −0.53, *p* < 0.001), lower child BMI centile (*b* = −0.41, *se* = 0.23, *β* = −0.21, *p* = 0.08), lower food responsiveness (*b* = −3.06, *se* = 1.70, *β* = −0.22, *p* = 0.08), and lower satiety responsiveness (*b* = −4.78, *se* = 2.38, *β* = −0.31, *p* = 0.05).

For energy intake the final model accounted for 47% of variance (R^2^ = 0.65, F = 11.05, *p* < 0.001). Significant predictors included the intervention group (reduction or replacement), baseline energy intake, and food responsiveness. Higher energy intake in week 3 was associated with being in the reduction group (*b* = −125.83, *se* = 58.49, *β* = −0.26, *p* = 0.04), having a higher baseline energy intake (*b* = 0.71, *se* = 0.13, *β* = 0.72, *p* < 0.001) and lower food responsiveness (*b* = −18.05, *se* = 7.85, *β* = −0.29, *p* = 0.03).

For fat intake, the final model accounted for 49% of variance (R^2^ = 0.49, F = 6.88, *p* < 0.001). Significant predictors included intervention group (reduction or replacement), baseline fat intake, and child age. Non-significant predictors included food responsiveness and deprivation score^1^. Higher intake of fat in week 3 was associated with being in the reduction group (*b* = −10.44, *se* = 3.28, *β* = −0.40, *p* = 0.03), having a higher baseline energy intake (*b* = 0.71, *se* = 0.15, *β* = 0.58, *p* < 0.001), being older (*b* = 0.33, *se* = 0.16, *β* = 0.25, *p* = 0.001), and scoring lower on food responsiveness (*b* = −0.83, *se* = 0.41, *β* = −0.25, *p* = 0.05).

For sugar intake, the final model accounted for 55% of variance (R^2^ = 0.55, F = 11.29, *p* < 0.001). Significant predictors included intervention group (reduction or replacement), baseline sugar intake, and deprivation score^1^. Higher intake of sugar in week 3 was associated with being in the reduction group (*b* = −16.09, *se* = 6.05, *β* = −0.31, *p* = 0.01), reporting a higher baseline sugar intake (*b* = 0.66, *se* = 0.12, *β* = 0.66, *p* < 0.001), having a higher deprivation score (higher scores indicate lower levels of deprivation) (*b* = 1.95, *se* = 0.95, *β* = 0.24, *p* < 0.05) and scoring high on food fussiness (*b* = 1.04, *se* = 0.61, *β* = −0.19, *p* = 0.10).

### 3.6. Longer Term Effects of the Intervention on Snack Frequency (4–6 Weeks Follow-Up)

Responses from the FFQ identified no significant changes to the frequency of snack intake pre and post intervention diet (*p* > 0.05), despite the majority of mothers expressing in interviews and the follow-up questionnaire that the intervention had impacted their habitual feeding practices and child’s nutritional intake (Table 5). Interview responses were categorised into two subthemes reflecting these experiences (Table A2).

#### 3.6.1. Theme 1: Change to Habitual Feeding Practices

Mothers reported that participation in the intervention resulted in them thinking more about the type of food to offer their child at meal and snack occasions. In particular, mothers focused on increasing fruit and vegetable offerings to enhance diet variety. Mothers also discussed limiting intake of HED snacks and availability of these items in the home. “*The study helped me think more about what he was eating and whether he needed snacks. Also, it has made me focus on his main meals more to keep them more balanced and healthy*” (P261, Reduction, male, 56 months).

#### 3.6.2. Theme 2: Impact on Consumption

Six weeks post intervention, mothers reported noticing that their children were more accepting of novel food items and since being offered more fresh fruit and vegetable snacks, they were consuming more as part of the habitual diet. Mothers reported that their children’s eating behaviours at meal times were also noticeably better, with more being eaten and less waste. Some mothers also felt that taking part in the intervention had a positive impact on the dietary intake of other family members, including themselves (mother) and the child’s siblings. Others reported no differences to their habitual diet. “*His sister now eats similar snacks to him and will ask for things like peppers rather than fruit*” (P104, Replacement, male, 50 months).

## 4. Discussion

The current pilot study aimed to explore the feasibility and acceptability of two strategies of snack portion control and examine the efficacy of the two methods to improve the habitual diets of preschool children. The results suggest that the study fulfilled the predefined feasibility and acceptability objectives. Whilst both interventions were rated positively, more mothers rated the replacement strategy as acceptable despite acknowledging that more preparation effort was required. Additionally, the secondary aim of testing the preliminary efficacy of the two interventions on dietary intake demonstrated the potential benefits of the replacement strategy when compared to the snack reduction strategy. Vegetable intake was higher in the replacement group compared to the reduction group, total energy (kcal/day), sugar (g) and fat (g) intakes were also decreased in the replacement strategy. Regardless of the apparent benefits of the replacement strategy, overall mothers reported that taking part in the study had prompted them to think about the snacks that they offer their children with a view to reducing HED snack intake. Overall, the findings of this pilot study are useful for informing the development of a larger trial.

The study provided evidence for identifying, recruiting and retaining parent-child dyads for a three-week intervention within the home environment. Once participants had been randomised into the intervention period, compliance rates were moderate with 72% of mothers following the intervention schedule at least 50% of the time. All mothers that were recruited completed the study and only one mother was removed from the analysis due to not returning the final food diary.

The ratings of study procedures were examined and were overall found to be acceptable. Participation was high, supporting the notion that four days is a suitable duration to record in the home environment, and anything above this threshold might result in unsatisfactory reporting and participant burnout [56]. Weighed food diaries have been found to be more accurate than recall methodologies however, mothers reported that they had to rely on recall and estimation at times. To facilitate record keeping, many parents took photos of their child’s food and drink items to prevent having to rely on memory. New technologies have been developed through mobile applications to support better estimation of portion size [57,58]. These technologies were not used in the present study however, they may be considered for future work as the portion size estimations that are produced are highly correlated with weighed foods [59].

Snack provision and snacking schedules were implemented to standardise exposure across all participants as much as possible and to assess the effects on dietary intake. In week 2 when all HED snacks were replaced for all children, mothers reported that the snacks were suitable, well liked, and similar to what their child usually consumed. In week 3, there were mixed responses regarding type (replacement) and quantity (reduction) of snacks provided. In the replacement condition, most children accepted the fruit and vegetable snacks if they were relatively liked and familiar. However, when children reported not liking the items offered, they refused these snacks and asked for alternative snacks, and this helps to explain the differing levels of compliance. In the reduction condition, most children accepted the reduced snack portion size and most parents complied with providing 50% of the snack. Parents are therefore willing and able to adhere to recommendations by PHE to provide 100 kcal snacks, and no more than two per day [19]. Even if the child consumed three snacks per day, total snack intake averaged less than 200 kcal in the replacement condition.

At times, children noticed when snacks were smaller than normal, and this shows that young children learn through exposure and social learning what amounts of foods to expect [25]. When snacks were offered on plates and bowls, portion size judgements are more difficult and therefore changes to portion size are less noticeable [60]. Exposure to packaging may create a portion size norm, which often is too large for young children and it may lead to overconsumption of items that are high in sugar and energy [61]. Large portion sizes have become normalised [62], such that consumers no longer perceive themselves to be overconsuming, and this seems to hold true for some of the children in the current study who rejected the 50% portion from the package. Recent work on adults [63] demonstrates that portion sizes can be relearned or “recalibrated” whereby following multiple exposures adults learn to accept a smaller portion size as being “normal”. However, to date this has not been investigated in children.

Preliminary efficacy analysis indicates that snack replacement improved dietary intake when compared to snack reduction. Vegetable intake was significantly increased in the replacement group compared to the reduction intervention. Offering vegetables as a snack increased total intake but did not displace vegetable intake at meal times. This finding confirms those reported by Roe et al. [26] demonstrating that when a variety of vegetables was served to preschool children in a child care environment vegetable intake increased. In the current study, overall intake of fruit was higher when compared to vegetables and there were no significant differences between intervention groups. Total fruit intake and fruit snack intake was higher in the intervention week (week 3) compared to weeks 1 and 2. This result appears to be driven by the trends observed in the replacement group; fruit intake increased in the replacement group in week 3 by around 20 g/day compared to week 1. This trend was not observed in the reduction group.

In the replacement group, total energy intake (kcal) was lower in week 3 compared to weeks 1 and 2 by around 145 kcal/day and 87 kcal/day respectively. This effect was not observed in the reduction group. Incorporating LED foods in to the habitual diet has robustly been demonstrated in children and adults to be effective at reducing total energy intake [59,64,65]. It is likely that the addition of extra vegetables and fruit accounts for this reduction in total daily energy intake. Alternatively, this may be attributable to reductions in total fat and sugar intake.

The results of the current investigation suggest that snack replacement compared to snack reduction is better aligned with Public Health England [19] and the World Health Organisation [22] sugar reduction aims. Both interventions were designed with the potential to reduce sugar intake. Whilst an overall main effect of study week was observed, trends in total and free sugar intake revealed that in the replacement group intake of total sugar in the intervention week (week 3) declined by around 17 g/day and free sugar by 15 g/day as compared to baseline (week 1). The magnitude of change was not as large in the reduction group. Total sugar declined by around 3.5 g/day and free sugar by around 8 g/day in the intervention week compared to baseline.

Despite the study not being sufficiently powered to detect conclusive effects of snack reduction or replacement on habitual diet, this pilot study does demonstrate clear advantages of the replacement strategy and more importantly that the necessary data could be collected. Despite the increased preparation required in the replacement strategy more mothers reported that they were content to continue with this strategy compared to snack reduction.

The target sample of 46 was successfully achieved by over recruiting to account for withdrawal and participants who may not have been eligible. Approximately half of the sample was residing in one of the 50% most deprived neighbourhood areas in Sheffield, suggesting that identifying, visiting, and building rapport with toddler group leaders and attendees in multiple areas of lower socioeconomic status is a suitable method to recruit under researched populations, who tend to be at greater risk of obesity [66]. However, only a minority of the children was from low-income families, with the majority earning more than the average UK household income. Future studies should explore the effects of portion size reduction strategies in lower income populations. Evidence suggests that consumption of HED snacks is inversely related to socioeconomic position [67]. Recent evidence from the UK suggests that mothers from more deprived backgrounds are more likely to offer young children HED “treat” foods compared to mothers from a higher socioeconomic position [68]. Data from the US demonstrates a “non-nutritive” role of snacks in lower income as compared to more affluent families [69], in that HED snacks are often used to modify children’s behaviours and more importantly they are not perceived as foods per se [70]. Elevated intakes of inexpensive HED snacks consumed from a young age may contribute in part to inequalities in health.

The results of the current study demonstrate the greatest improvement to children’s diets were in the replacement condition, regardless of differences at baseline. For example, the replacement group consumed less fruit and vegetables as snacks, more total energy, sugar, and fat. Moreover, the replacement group was disadvantaged in terms of employment and education, and this group had a higher average child and maternal BMI. It is well documented that a healthful diet is more costly compared to a diet containing more HED foods [71,72]. Furthermore, food waste is an important issue that needs to be carefully considered. For example, children in the replacement intervention group were offered between 40–120 g of vegetables per day depending upon how many snacks they consumed, yet an average increase of 20 g/day was observed thus resulting in a significant and costly amount of waste. Replacement snacks were more expensive than HED snacks (£11.59 versus £4.11), and children produced more food waste in the replacement (366 g = 51%) versus reduction group (27 g = 15%). More research is required to examine this further.

Efforts to standardise snack intake across the participant pool resulted in unintended consequences of increasing snack intake in week 2 as compared to week 1, and providing snack options that some children rejected. In future, parents should select snacks (both HED and fruits and vegetables snacks) that are habitually eaten and well-liked by their children on an individual rather than group level. A second limitation of the study was the exclusion of children attending childcare for more than three full consecutive days. This criterion implies that the study may not be feasible to all cohorts of preschool children, which limits the generalizability of the findings and the impact of the current design.

It is not known whether either intervention would be sustainable over a longer period and so longer-term research is needed. At 4–6 weeks follow up some mothers reported that they felt that engaging in the study, regardless of group assignment, had positively influenced the snacks that they offered their children in that they were offering more fruits and vegetables. Yet, the results of the FFQ did not support this, an increase in the frequency of offering fruit and vegetables was not detected. Individual difference in response to portion sizes have been documented [73], increasing the number of participants in future investigations would allow researchers to further explore how eating traits and family circumstances might impact the success of the intervention. Characterisation of individual differences in response to portion size will aid the development of successful interventions.

## 5. Conclusions

This study is the first to explore the feasibility and acceptability of two portion control strategies for snacks in UK-based preschool children in the home environment. Snack reduction and snack replacement appear to be feasible methods of portion control in the home environment. The current study demonstrates that the recruitment strategy, retention rates, and methods of data collection were acceptable; however, the replacement strategy appeared to be associated with more dietary improvement than reduction. Mothers reported being content with the replacement strategy; children’s vegetable intake increased and fat intake decreased. The results of this pilot study highlight issues for intervention refinement and provide important feasibility, acceptability, and preliminary efficacy information necessary to design a larger and more adequately powered trial.

## Figures and Tables

**Table 1:** Nutritional composition of snacks included in week 2 and 3 snacking schedules (per portion).

	Weight/Portion (g)	Energy (kcal)	Energy Density (kcal/g)	Fat (g)	Carbohydrate (g)	Sugar (g)	Salt (g)
Chocolate coated sponge cookie (Jaffa Cakes, McVitie’s ^®^)	37	144	3.9	3.6	25.8	19.2	0.07
Cookies (Digestives, McVitie’s ^®^)	30	142	4.7	6.4	18.6	5	0.4
Ritz Crackers (Mondelēz ©)	32	146	4.6	5.7	20.5	2.9	0.7
Oat-bar (Goodies, Organix ^®^)	30	121	4.0	4.5	17	7.8	0.01
Yoghurt coated raisins (Sunny Raisins, Whitworths)	25	112	4.5	5.1	15.8	15.8	0
Cheese flavoured crackers ( Mini Cheddars, Jacobs ^®^)	25	128	5.1	7.3	12.5	1.3	0.6
Salted potato chips (Pom-Bear, Intersnack ©)	15	79	5.3	4.2	9.6	0.5	0.26
Bell Pepper (Red, yellow and orange)	40	11	0.3	0.08	2.53	0	0
Grapes (White)	40	28	0.7	0.06	7.24	6.2	0
Apple (Gala)	40	20	0.5	0.25	5.25	4	0
Banana	40	36	0.9	0.1	9	4.9	0
Carrot	40	16	0.4	0.1	3.8	1.8	0
Cucumber	40	6	0.2	0.04	1.45	0.7	0
Pear	40	23	0.6	0	6	4	0.05
Tomato (cherry)	40	7	0.2	0.08	1.57	1.1	0
Rice cake (Kallø)	7	30	4.3	0.2	6.3	0.1	0.1
Breadstick (Tesco own brand)	8.25	34.5	4.2	0.6	6	0.3	0.15
Crackerbread (Ryvita ^®^)	7.5	27	3.6	0.15	5.7	0.15	0.1

**Table 2:** Demographic information for mother-child dyads (mean ± SD).

Child	Total *n* = 46	Reduction *n* = 22	Replacement *n* = 24
Gender	52% male	63% male	39% male
Age (months)	36.6 ± 9.5	35.8 ± 9.9	37.5 ± 8.9
BMI Centile	60.9 ± 26.7	56.0 ± 30.2	66.2 ± 21.8
Mother			
Age (years)	35 ± 4	35 ± 3	35 ± 5
* BMI (kg/m^2^)	24.7 ± 5.2	23.2 ± 3.5	26.3 ± 6.2
Ethnicity	White British, mixed or other 94% Chinese 4% Asian Indian 2%	White British, mixed or other 96% Chinese 4%	White British, mixed or other 92% Chinese 4% Asian Indian 4%
Highest education	>A-level or equivalents 74%	>A-level or equivalents 88%	>A-level or equivalents 61%
Employment Status	63% Employed full/part time or on maternity leave	71% Employed full/part time or on maternity leave	57% Employed full/ part time or on maternity leave
Residential Status	Own with or without mortgage 78%	Own with or without mortgage 88%	Own with or without mortgage 65%
Marital Status	100% married or cohabiting	100% married or cohabiting	100% married or cohabiting
Income	£0–10,000 4%	£0–10,000 5%	£0–10,000 4%
	£10–20,000 28%	£10–20,000 32%	£10–20,000 25%
	£20–30,000 22%	£20–30,000 23%	£20–30,000 21%
	£30–40,000 24%	£30–40,000 23%	£30–40,000 25%
	£40,000+ 22%	£40,000+ 18%	£40,000+ 25%

* Body mass index (BMI).

**Table 3:** Nutritional intake per day in week 1, 2, and 3 (mean ± SD).

	Reduction			Replacement			Total		
	Week1	Week2	Week3	Week1	Week2	Week3	Week1	Week2	Week3
Vegetable: Snacks (g)	1.7 ± 3.9	1.3 ± 3.0	0.5 ± 1.8	0.2 ± 0.6	0.6 ± 2.3	21.0 ± 21.8 *^#^	0.9 ± 2.8	0.9 ± 2.7	11.2 ± 18.8 *^#^
Vegetable: Meals (g)	24.8 ± 19.1	19.6 ± 12.0	20.3 ± 17.0	28.5 ± 29.8	24.3 ± 23.6	24.9 ± 23.0	26.8 ± 25.0	22.0 ± 18.9	22.7 ± 20.3
Vegetable: Total (g)	26.5 ± 20.3	20.9 ± 12.5	20.8 ± 17.0	28.7 ± 29.8	24.9 ± 24.9	45.9 ± 35.1 *^#^	27.7 ± 25.4	23.0 ± 19.8	33.9 ± 30.5 ^#^
Fruit: Snacks (g)	65.6 ± 75.7	45.1 ± 31.4	65.9 ± 50.8	42.0 ± 31.6	27.6 ± 31.1	65.4 ± 41.6	53.3 ± 57.7	36.0 ± 32.1 *^#^	65.6 ± 45.7
Fruit: Meals (g)	34.4 ± 42.0	39.1 ± 33.3	37.1 ± 36.4	36.7 ± 28.1	36.7 ± 27.4	33.5 ± 31.3	35.6 ± 35.1	37.8 ± 30.0	35.2 ± 33.5
Fruit: Total (g)	100.0 ± 71.8	84.2 ± 40.4	102.9 ± 63.0	78.7 ± 46.5	64.3 ± 49.7	99.0 ± 51.8	88.9 ± 60.2	73.8 ± 46.1	100.9 ± 56.8 ^#^
Energy (kcal)	1052.1 ± 235.8	1077.8 ± 229.1	1063.5 ± 284.1	1116.3 ± 239.6	1058.5 ± 225.2	971.8 ± 188.3 *^#^	1085.6 ± 37.3	1067.7 ± 224.7	1015.7 ± 240.7
Total Sugar (g)	71.1 ± 21.9	69.9 ± 19.6	67.5 ± 23.7	79.7 ± 28.2	69.8 ± 19.5	62.6 ± 26.6	75.6 ± 25.5	69.9 ± 19.3	65.0 ± 25.1 *
Free Sugar (g)	29.2 ± 15.4	24.3 ± 17.0	20.8 ± 13.0	40.4 ± 26.7	27.2 ± 14.7	25.2 ± 24.6	35.1 ± 22.5 ^#^	25.8 ± 15.7	23.1 ± 19.8 *
Total Fat (g)	38.2 ± 9.1	42.8 ± 10.2	41.9 ± 15.8	42.0 ± 11.5	42.4 ± 12.6	34.6 ± 9.2 *^#^	40.2 ± 10.5	42.6 ± 11.4	38.1 ± 13.2 ^#^
Mean number of snacks	1.6 ± 0.6	2.1 ± 0.6	2.1 ± 0.6	2.0 ± 0.6	2.2 ± 0.5	2.1 ± 0.5	1.8 ± 0.6 ^#^	2.1 ± 0.5	2.1 ± 0.6 *

Results from one-way repeated measure ANOVA. * significantly different to week 1. ^#^ significantly different to week 2.

**Table 4:** Predictors of vegetable intake in week 3: output from a linear regression.

	Vegetables				Fresh Fruit				Total Energy				Total Fat				Total Sugar			
	*b*	*se*	*β*	*p*	*b*	*se*	*β*	*p*	*b*	*se*	*β*	*p*	*b*	*se*	*β*	*p*	*b*	*se*	*β*	*p*
Intervention Group	23.91	6.34	0.39	0.001	23.81	12.42	0.22	0.06	−125.83	58.49	−0.26	0.04	−10.44	3.28	−0.40	<0.01	−16.09	6.05	−0.31	0.01
Baseline Intake	0.72	0.13	0.58	<0.001	0.72	0.13	0.63	<0.001	0.71	0.13	0.72	<0.001	0.71	0.15	0.58	<0.001	0.66	0.12	0.66	<0.001
Child Neophobia	−1.59	0.62	−0.27	0.01	−7.99	2.21	−0.77	<0.01	-	-	-	-	-	-	-	-	-	-	-	-
Deprivation Score *	2.06	0.98	0.21	0.04	-	-	-	-	-	-	-	-	0.88	0.51	0.22	0.09	1.95	0.95	0.24	<0.05
Food Fussiness	-	-	-	-	5.84	2.56	0.53	0.03	-	-	-	-	-	-	-	-	1.04	0.61	0.19	0.10
Modelling	-	-	-	-	−10.25	2.38	−0.53	< 0.001	-	-	-	-	-	-	-	-	-	-	-	-
Food Responsiveness	-	-	-	-	−3.06	1.70	−0.22	0.08	−18.05	7.85	−0.29	0.03	−0.83	0.41	−0.25	0.05	-	-	-	-
Satiety Responsiveness	-	-	-	-	−4.78	2.38	−0.31	0.05	-	-	-	-	-	-	-	-	-	-	-	-
* BMI Centile	-	-	-	-	−0.41	0.23	−0.21	0.08	-	-	-	-	-	-	-	-	-	-	-	-
Child Age	-	-	-	-	-	-	-	-	-	-	-	-	0.33	0.16	0.25	< 0.05	-	-	-	-

* Higher scores indicate lower levels of deprivation. * Body mass index (BMI).

**Table 5:** Frequency of consumption pre and post intervention (mean ± SD).

Food Item	Pre-Intervention			Post Intervention		
	Reduction	Replacement	Total	Reduction	Replacement	Total
Cookies	5.68 ± 4.98	2.96 ± 2.03	4.26 ± 3.95	3.55 ± 2.32	2.92 ± 2.59	3.18 ± 2.47
Cake	2.36 ± 2.75	1.49 ± 2.06	1.91 ± 2.43	1.66 ± 1.70	1.14 ± 1.07	1.36 ± 1.37
Pastries	0.34 ± 0.47	0.28 ± 0.31	0.31 ± 0.39	0.30 ± 0.32	0.16 ± 0.24	0.22 ± 0.28
Sweets	3.64 ± 3.11	2.52 ± 2.88	3.03 ± 3.00	3.38 ± 3.64	2.09 ± 1.95	2.63 ± 2.82
Potato Chips	3.56 ± 2.69	2.40 ± 2.07	2.93 ± 2.42	3.13 ± 2.18	2.76 ± 2.97	2.91 ± 2.64
Green cooked vegetables	6.61 ± 4.97	4.78 ± 3.01	5.66 ± 4.12	6.27 ± 5.13	5.50 ± 4.60	5.82 ± 4.78
Other Vegetables	4.59 ± 3.30	4.06 ± 3.04	4.32 ± 3.14	4.66 ± 3.13	3.98 ± 2.24	4.26 ± 2.64
Salad	4.69 ± 4.07	2.68 ± 2.24	3.64 ± 3.37	4.20 ± 3.42	3.86 ± 4.45	4.01 ± 4.00
Fruit	13.14 ± 8.59	11.42 ± 5.32	12.24 ± 7.05	13.69 ± 6.79	14.73 ± 7.62	14.29 ± 7.21

## Appendix

**Table A1:** Acceptability Questionnaire.

1. Please circle the group you were assigned to in the study				
Reduction			Replacement	
THINK ABOUT THE SECOND WEEK OF THE STUDY WHEN WE PROVIDED THE SNACKS FOR YOUR CHILD				
2. The type of snacks provided in the snack pack for week two were appropriate for my child				
Strongly disagree	Disagree	Neither agree nor disagree	Agree	Strongly Agree
3. My child liked the snacks in week two				
Strongly disagree	Disagree	Neither agree nor disagree	Agree	Strongly Agree
4. The snacks offered during this week were similar to the snacks my child would normally eat				
Strongly disagree	Disagree	Neither agree nor disagree	Agree	Strongly Agree
THINK ABOUT THE THIRD WEEK OF THE STUDY WHEN WE ASKED YOU TO REPLACE OR REDUCE YOUR CHILD’S SNACKS				
5. My child’s hunger was satisfied by the snacks in week three				
Strongly disagree	Disagree	Neither agree nor disagree	Agree	Strongly Agree
6. My child was happy with the snacks in week three				
Strongly disagree	Disagree	Neither agree nor disagree	Agree	Strongly Agree
7. My child noticed the changes to his/her snacks				
Strongly disagree	Disagree	Neither agree nor disagree	Agree	Strongly Agree
8. My child noticed the changes to his/her drinks				
Strongly disagree	Disagree	Neither agree nor disagree	Agree	Strongly Agree
9. Keeping the food diary was inconvenient				
Strongly disagree	Disagree	Neither agree nor disagree	Agree	Strongly Agree
10. Keeping the food diary was difficult				
Strongly disagree	Disagree	Neither agree nor disagree	Agree	Strongly Agree
11. Keeping the food diary was helpful				
Strongly disagree	Disagree	Neither agree nor disagree	Agree	Strongly Agree
12. Whilst keeping the food diary I chose different foods in order to make record keeping easier				
Strongly disagree	Disagree	Neither agree nor disagree	Agree	Strongly Agree
13. How willing would you be to use this method to reduce your child’s portion sizes?				
Very unwilling	Unwilling	Neither willing nor unwilling	Willing	Very willing
14. How likely is this method to make permanent changes to your child’s eating habits?				
Very unlikely	Unlikely	Neither likely nor unlikely	Likely	Very likely
15. I found it easy to change my child’s snacks				
Strongly disagree	Disagree	Neither agree nor disagree	Agree	Strongly Agree
16. I found it easy to change my child’s drinks				
Strongly disagree	Disagree	Neither agree nor disagree	Agree	Strongly Agree

**Table A2:** Quotations supporting the themes constructed from interviews with mothers about their experiences taking part in the intervention.

Theme	Sub Theme	Supporting Quotations
1: Reasons for non-compliance	1.1 In the care of others	“*Nursery aren’t going to follow the plan as the management aren’t happy with the snacks*” (P190, Reduction, male, 22 months)
		“*some days at nursery she didn’t want what she was having in her bag but I told them that she’s not meant to be isolated with it*” (P77, Replacement, female, 49 months)
		“*I felt like sometimes I didn’t want to put too much imposition on them, I felt sorry for my mother-in-law having to deal with him screaming*” (P214, Replacement, male, 30 months)
		“*My mum and dad are terrible with him, giving him chocolate and things like that and my husbands a nightmare, like he gave him a mars bar from a celebration pack yesterday morning for breakfast and I was fuming because he knows that he can’t have that*” (P74, Replacement, male, 30 months)
		“*In the morning he asked for snack and his dad gave the whole pack of jaffa cakes*” (P2, Reduction, male, 39 months)
	1.2 Children’s health and behaviour	“*He’s been ill; it has been really quite tricky because his appetite is not right. I want him to eat so I am more like have whatever you want. I was like you want crisps go get crisps*” (P2, Reduction, male, 39 months)
		“*The only problems I guess was when he was ill because it was hard to, because he wasn’t eating as normal. Trying to get him to eat, because he just didn’t want to*” (P202, Replacement, male, age 29 months)
		“*She’s been crying, not happy, upset, so I’ve been giving her more tasty or unhealthy snack to be able to manage her behaviour. I gave her cookie at the doctors as she was upset*” (P84, Reduction, female, 37 months)
		“*I just gave it to him. I said this is what you’ve got we are going to V club in half an hour, you either eat it or you don’t*” (P104, Replacement, male, age 50 months
		“*I just stuck to my guns and said no you’re not having it. I mean it’s hard at the time but I stuck to my guns*” (P34, Replacement, female, 48 months)
	1.3 Organisation	“*it was okay because I just did it all at the beginning of the week, it felt a bit strange obviously getting rid of half of it, but mm it was okay. I was just more organised. I think by this stage I had cracked it*” (P148, Reduction, female, 26 months)
		“*It was kind of helpful to be prompted to be organised. so I would get everything ready the night before, so sometimes I would split one thing into two bags and then I would have another days bag full all ready to go, and that was really convenient*” (P205, Reduction, female, 29 months)
		“*I was quite often forgetting to give half. With pom bears I gave her the pack forgetting that it should be half.*” (P84, Reduction, female, 37 months)
2: Acceptability	2.1 Recording in the food diary	“*I found it absolutely fine, it was just a case of remembering to weigh everything, but the instructions on how to do it was clear*” (P199, Reduction, female, 34 months)
		“*easy, it was easy peasy. I just got it into my routine. I just wrote it every time, every meal, I wrote everything straight away, I weighed it, wrote it down, served it and then weighed what was left*” (P33, Reduction, female, 39 months)
		“*it was just obviously when out and about when I didn’t have the scales it became a bit trickier because I realised I have no idea about how much things weigh at all*” (P143, Replacement, male, 46 months)
	2.2 Snack Type	“*I think that was fairly standard but then I think this week wasn’t all that dissimilar to what I would have been doing anyway*” (P291, Replacement, female, 26 months)
		“*I don’t think she cared really actually as long as she likes it she’ll eat it. She wasn’t asking for anything any different”.* (P160, Replacement, female, 28 months)
		“*Pear, he wouldn’t touch pear, I tried him with the skin on, without the skin, I did all that with him”.* (P74, Replacement, boy, age 30 months).
	2.3 Snack preparation and serving method	“*it was obviously a little bit more faffy than the other one because you have to weigh it, erm, washing it and prepping it before you go out and stuff like that*” (P132, Replacement, male, 45 months)
		“*Like the crisps maybe I put them in a bowl or something like that so maybe that’s why she didn’t notice as much*” (P20, Reduction, female, 52 months)
		“*I kind of tried to serve the half serving in the packet although she did question to where the other half was erm, I took half of them out and she knew then, she was like ‘I want more, there’s more’ so I gave her another one and she was okay*” (P199, Reduction, female, 34 months).
	2.4 willingness to continue the intervention	“*I will be carrying on and giving her, I’ll mix it all up and make sure I am offering more fruit and veg snacks definitely*” (P77, Replacement, female, 49 months)
		“*if you said you could give him anything as long as you give him half portions that would be fine with me, but giving him just these snacks (in the schedule), I don’t think I’d be able to do it*” (P190, Reduction, male, 22 months)
3. Longer term effects of the intervention	3.1 Changes to habitual feeding practices	“*The study helped me think more about what he was eating and whether he needed snacks. Also it has made me focus on his main meals more to keep them more balanced and healthy*” (P261, Reduction, male, 56 months)
		“*The combinations of food I give as snacks has changed. I think it has introduced more variety. I now buy crackers, rather than crisps so often, and I give more vegetable snacks than before*” (P104, Replacement, male, 50 months)
	3.2 Impact on consumption	“*He is more willing to try other items, but that could be because I’ll offer different options over favourites*” (P234, Replacement, male, 24 months)
		“*She is tending to finish snacks and meals more often and waste less food*” (P142, Reduction, female, 39 months)
		“*My 6year old now eats more fruit as a snack too*” (P291, Replacement, female, 26 months)
		“*His sister now eats similar snacks to him and will ask for things like peppers rather than fruit*” (P104, Replacement, male, 50 months)
		“*no, we have a food routine which we went back to*” (P208, Replacement, male, 26 months)
		*”No, she’s continued to have the same amount of snacks*” (P199, Reduction, female, 34 months)
